# Bis(1,1-dimethyl­guanidinium) tetra­aqua­dimethyl­tin(IV) bis­(sulfate)

**DOI:** 10.1107/S1600536811053761

**Published:** 2011-12-21

**Authors:** Mouhamadou Sembene Boye, Aminata Diasse-Sarr, Eric Lebraud, Philippe Guionneau

**Affiliations:** a Departement de Chimie, Faculté de Sciences et Techniques, Universite Cheikh Anta, Diop, Dakar, Senegal; b CNRS, Universite de Bordeaux, ICMCB, 87 avenue du Dr A. Schweitzer, Pessac F-33608, France

## Abstract

Single crystals of the title salt, (C_3_H_10_N_3_)_2_[Sn(CH_3_)_2_(H_2_O)_4_](SO_4_)_2_, formed concomitantly with the already known [Sn(CH_3_)_3_]_2_SO_4_·2H_2_O. In the title structure, the Sn^IV^ atom displays a slightly distorted octa­hedral coordination geometry defined by four O water atoms in the equatorial positions and two methyl groups in the axial positions. In the crystal, various O—H⋯O and N—H⋯O hydrogen-bonding inter­actions between the organic cation and the coordinated water mol­ecules as donors and the sulfate O atoms as acceptors result in a three-dimensional structure. The Sn^IV^ atom is located on an inversion centre, resulting in half of the complex metal cation being in the asymmetric unit.

## Related literature

For applications of tin-based materials, see: Molloy & Purcell (1986 ▶); Dutrecq *et al.* (1992 ▶); Gielen (2005 ▶). For oxoanion ligands, see: Boye & Diasse-Sarr (2007 ▶) and references therein. For [(Sn(CH_3_)_3_)_2_SO_4_]^.^2H_2_O, see: Molloy *et al.* (1989 ▶).
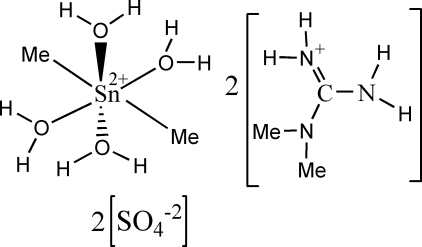


## Experimental

### 

#### Crystal data

(C_3_H_10_N_3_)_2_[Sn(CH_3_)_2_(H_2_O)_4_](SO_4_)_2_
*M* *_r_* = 589.26
Monoclinic, 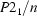

*a* = 6.683 (1) Å
*b* = 12.609 (2) Å
*c* = 13.469 (2) Å
β = 99.207 (10)°
*V* = 1120.4 (3) Å^3^
*Z* = 2
Mo *K*α radiation
μ = 1.39 mm^−1^
*T* = 293 K
0.25 × 0.12 × 0.10 mm

#### Data collection

Nonius KappaCCD diffractometer
Absorption correction: multi-scan (*SCALEPACK*; Otwinowski & Minor, 1997 ▶) *T*_min_ = 0.722, *T*_max_ = 0.873
13961 measured reflections
4462 independent reflections
3306 reflections with *I* > 2σ(*I*)
*R*_int_ = 0.048

#### Refinement

*R*[*F* ^2^ > 2σ(*F* ^2^)] = 0.034
*wR*(*F* ^2^) = 0.084
*S* = 1.07
4462 reflections
168 parameters
H atoms treated by a mixture of independent and constrained refinement
Δρ_max_ = 1.51 e Å^−3^
Δρ_min_ = −1.17 e Å^−3^

### 

Data collection: *COLLECT* (Nonius, 2003 ▶); cell refinement: *SCALEPACK* (Otwinowski & Minor, 1997 ▶); data reduction: *DENZO* (Otwinowski & Minor, 1997 ▶); program(s) used to solve structure: *SHELXS97* (Sheldrick, 2008 ▶); program(s) used to refine structure: *SHELXL97* (Sheldrick, 2008 ▶); molecular graphics: *ORTEP-3* (Farrugia, 1997 ▶); software used to prepare material for publication: *publCIF* (Westrip, 2010 ▶).

## Supplementary Material

Crystal structure: contains datablock(s) I, New_Global_Publ_Block. DOI: 10.1107/S1600536811053761/wm2575sup1.cif

Structure factors: contains datablock(s) I. DOI: 10.1107/S1600536811053761/wm2575Isup2.hkl

Additional supplementary materials:  crystallographic information; 3D view; checkCIF report

## Figures and Tables

**Table 1:** Selected bond lengths (Å)

Sn1—C1	2.094 (2)
Sn1—O3	2.2140 (16)
Sn1—O2	2.2240 (17)

**Table 2:** Hydrogen-bond geometry (Å, °)

*D*—H⋯*A*	*D*—H	H⋯*A*	*D*⋯*A*	*D*—H⋯*A*
O2—H22⋯O13^i^	0.79 (4)	1.90 (4)	2.680 (2)	169 (4)
O2—H12⋯O12^ii^	0.72 (4)	1.91 (4)	2.627 (2)	175 (4)
O3—H23⋯O11^iii^	0.73 (3)	1.91 (3)	2.629 (3)	168 (3)
O3—H13⋯O10^iv^	0.91 (3)	1.71 (3)	2.615 (2)	172 (3)
N2—H2*N*⋯O13^v^	0.79 (3)	2.10 (3)	2.887 (2)	174 (3)
N3—H3*N*⋯O12^v^	0.87 (3)	2.04 (3)	2.899 (3)	172 (2)
N2—H1*N*⋯O10^iv^	0.82 (3)	2.14 (3)	2.918 (3)	160 (2)
N3—H4*N*⋯O11^vi^	0.82 (3)	2.17 (3)	2.956 (3)	160 (3)

Symmetry codes: (i) 
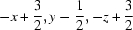
; (ii) 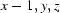; (iii) 
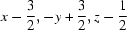
; (iv) 
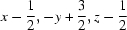
; (v) 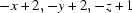; (vi) 
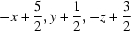
.

**Fig. 1.**
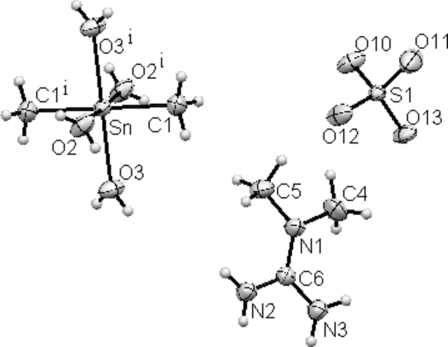
The molecular entities of the title compound showing the numbering scheme. Displacement ellipsoids are drawn at the 50% probability level. [Symmetry code i) -x+1, -y+1, -z+1.]

**Fig. 2.**
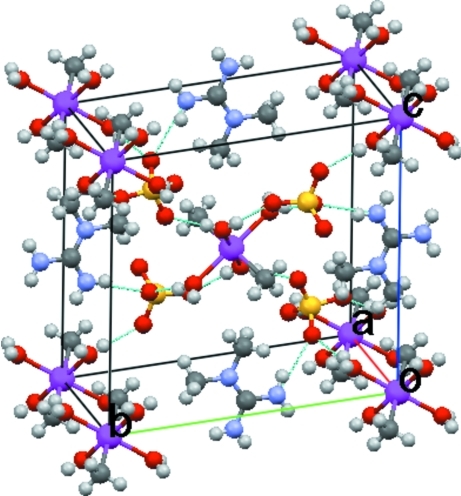
Molecular packing, showing intermolecular hydrogen bonding interactions (blue lines) as viewed along the a axis.

## supplementary crystallographic information

### Comment

Tin-based materials are known to exhibit a wide panel of potential applications in domains as different as plastic stabilizers, reaction catalysts (Molloy & Purcell, 1986), phytosanitory products (Dutrecq *et al.*, 1992) or medicinals (Gielen, 2005). In such context, any new tin-based molecular material description brings its contribution to the knowledge of organotin compounds diversity, revealing, for instance, the report of new crystal packings or coordination sphere geometries. To such aim, we focused on oxoanions, notably sulfate, to construct polymeric tin-based compounds (Boye & Diasse-Sarr, 2007). It has been previously reported that (Sn(CH_3_)_3_)_2_SO_4_^.^2H_2_O (Molloy *et al.*, 1989) crystallizes in an orthorhombic unit cell with a three-dimensional network based on an O_2_SnC_3_ coordination sphere, with the Sn^IV^ cation directly coordinated to the O atoms of the sulfate anion. Allowing dimethyl guanidinium sulfate to react which trimethyltin chloride, we obtained the known compound and concomitantly also single crystals of the title compound, (C_3_H_10_N_3_)_2_[Sn(CH_3_)_2_(H_2_O)_4_](SO_4_)_2_, the structure of which is described here. It is notable that these two compounds are optically undistinguishable and that all attempts gave mixtures of single crystals.

The asymmetric unit of the title compound contains  half of an [Sn(CH_3_)_2_(H_2_O)_4_]^2+^ cation with the Sn^IV^ cation located on an inversion centre, one dimethyl guanidinium cation and one sulfate anion. The Sn^IV^ cation is octahedrally coordinated to two methyl groups in axial and four oxygen atoms of water molecules in equatorial positions (Figure 1). This O_4_SnC_2_ environment constitutes the first major difference with the structure of the previously reported compound. The second one comes from the crystal packing itself (Figure 2), since sulfate anions are not coordinated to Sn. The O—S—O bonds angles [108.71 (11)° -110.27 (10)°] indicate a slight angular distorsion of the tetrahedral sulfate anion. The third major structural difference in comparison with the previously reported compound comes from the presence of additional dimethyl guanidinium cations. Dimethyl guanidinium and the complex metal cations are connected through hydrogen bonds to the sulfate O atoms (Table 2), resulting in a three-dimensional structure.

### Experimental

[Me_2_NC(=NH)NH_2_]_2_H_2_SO_4_ and SnMe_3_Cl are Aldrich chemicals used without further purification. The title derivative is obtained by mixing in an 1:1 ratio [Me_2_NC(=NH)NH_2_]_2_H_2_SO_4_ (0,61 g, 2,18 mmol) dissolved in methanol (40 ml) and a minimum of water (10 ml) and SnMe_3_Cl (0,43 g, 2,18 mmol) dissolved in dichloromethane. The mixture was stirred for around two hours at room temperature and by slow solvent evaporation gave prismatic crystals suitable for X-ray diffraction analysis. Apart from the title compound, (Sn(CH_3_)_3_)_2_SO_4_^.^2H_2_O crystals suitable for X-ray diffraction were also found in the same batch. The determined crystal structure is strictly the same as the one previously described. Crystals of both compounds are colourless (m.p.> 533 K) and present the same global shape and size. Only X-ray diffraction allowed to distinguish between these concomitant products.

The title compound was isolated according to the following reaction:

4[(Me_2_NC(=NH)NH_2_)_2_H_2_SO_4_] + 6SnMe_3_Cl + 8H_2_O →

[SnMe_2_(H_2_O)_4_](SO_4_)_2_[Me_2_NC(=NH_2_)NH_2_]_2_ + 2(SnMe_3_)_2_SO_4_.2H_2_O + 6[Me_2_NC(=NH_2_)NH_2_]Cl + SnMe_4_

### Refinement

H atoms of water and guanidinium were forund from Fourier synthesis and were refined freely. H atoms of the methyl groups were placed geometrically and were allowed for free rotation, with U_eq_(H) = 1.5U_eq_(C). The highest peak and the deepest hole in the final Fourier synthesis are 1.76 Å from O12 and 0.70 Å from Sn1, respectively.

### Crystal data

**Table:**

C_2_H_14_O_4_Sn·2(C_3_H_10_N_3_)·2(O_4_S)	*F*(000) = 604
*M**_r_* = 589.26	*D*_x_ = 1.747 Mg m^−^^3^
Monoclinic, *P*2_1_/*n*	Mo *K*α radiation, λ = 0.71069 Å
Hall symbol: -P 2yn	Cell parameters from 46680 reflections
*a* = 6.683 (1) Å	θ = 1.0–33.7°
*b* = 12.609 (2) Å	µ = 1.39 mm^−^^1^
*c* = 13.469 (2) Å	*T* = 293 K
β = 99.207 (10)°	Prism, colorless
*V* = 1120.4 (3) Å^3^	0.25 × 0.12 × 0.10 mm
*Z* = 2	

### Data collection

**Table:**

Nonius KappaCCD diffractometer	4462 independent reflections
Radiation source: fine-focus sealed tube	3306 reflections with *I* > 2σ(*I*)
graphite	*R*_int_ = 0.048
φ scans and ω scans with κ = 0	θ_max_ = 33.7°, θ_min_ = 3.5°
Absorption correction: multi-scan (*HKL**SCALEPACK*; Otwinowski & Minor, 1997)	*h* = −10→7
*T*_min_ = 0.722, *T*_max_ = 0.873	*k* = −19→19
13961 measured reflections	*l* = −20→21

### Refinement

**Table:**

Refinement on *F*^2^	Primary atom site location: structure-invariant direct methods
Least-squares matrix: full	Secondary atom site location: difference Fourier map
*R*[*F*^2^ > 2σ(*F*^2^)] = 0.034	Hydrogen site location: inferred from neighbouring sites
*wR*(*F*^2^) = 0.084	H atoms treated by a mixture of independent and constrained refinement
*S* = 1.07	*w* = 1/[σ^2^(*F*_o_^2^) + (0.0378*P*)^2^ + 0.1746*P*] where *P* = (*F*_o_^2^ + 2*F*_c_^2^)/3
4462 reflections	(Δ/σ)_max_ < 0.001
168 parameters	Δρ_max_ = 1.51 e Å^−^^3^
0 restraints	Δρ_min_ = −1.17 e Å^−^^3^

### Special details

**Table:**

Geometry. All s.u.'s (except the s.u. in the dihedral angle between two l.s. planes) are estimated using the full covariance matrix. The cell s.u.'s are taken into account individually in the estimation of s.u.'s in distances, angles and torsion angles; correlations between s.u.'s in cell parameters are only used when they are defined by crystal symmetry. An approximate (isotropic) treatment of cell s.u.'s is used for estimating s.u.'s involving l.s. planes.
Refinement. Refinement of *F*^2^ against ALL reflections. The weighted *R*-factor *wR* and goodness of fit *S* are based on *F*^2^, conventional *R*-factors *R* are based on *F*, with *F* set to zero for negative *F*^2^. The threshold expression of *F*^2^ > 2σ(*F*^2^) is used only for calculating *R*-factors(gt) *etc*. and is not relevant to the choice of reflections for refinement. *R*-factors based on *F*^2^ are statistically about twice as large as those based on *F*, and *R*– factors based on ALL data will be even larger.

### Fractional atomic coordinates and isotropic or equivalent isotropic displacement parameters (Å^2^)

**Table:**

	*x*	*y*	*z*	*U*_iso_*/*U*_eq_	
Sn1	0.5000	0.5000	0.5000	0.02430 (6)	
S1	1.36662 (7)	0.79887 (3)	0.78817 (3)	0.02461 (10)	
O3	0.4232 (3)	0.64310 (13)	0.40639 (14)	0.0413 (4)	
O2	0.3228 (3)	0.56250 (15)	0.61365 (13)	0.0411 (4)	
N3	0.7496 (3)	1.12233 (14)	0.49706 (16)	0.0375 (4)	
N2	0.7117 (3)	0.96848 (18)	0.40651 (15)	0.0356 (4)	
N1	0.7856 (3)	0.96105 (15)	0.58016 (13)	0.0342 (4)	
C6	0.7503 (3)	1.01623 (15)	0.49489 (16)	0.0280 (4)	
C5	0.7904 (4)	0.84580 (18)	0.58146 (19)	0.0426 (5)	
H5A	0.8283	0.8203	0.5199	0.064*	
H5B	0.6586	0.8191	0.5880	0.064*	
H5C	0.8875	0.8220	0.6373	0.064*	
C4	0.8083 (5)	1.0131 (2)	0.67791 (19)	0.0498 (7)	
H4A	0.7047	1.0660	0.6772	0.075*	
H4B	0.9392	1.0462	0.6919	0.075*	
H4C	0.7960	0.9615	0.7290	0.075*	
C1	0.7679 (3)	0.57383 (18)	0.56804 (17)	0.0379 (5)	
H1A	0.8647	0.5208	0.5949	0.057*	
H1B	0.7393	0.6190	0.6214	0.057*	
H1C	0.8223	0.6155	0.5190	0.057*	
O12	1.3577 (3)	0.75608 (13)	0.68640 (12)	0.0480 (5)	
O11	1.5624 (3)	0.77413 (14)	0.84837 (16)	0.0582 (5)	
O13	1.3383 (3)	0.91454 (10)	0.78006 (11)	0.0377 (3)	
O10	1.2059 (3)	0.75053 (12)	0.83607 (13)	0.0428 (4)	
H22	0.263 (6)	0.524 (3)	0.645 (3)	0.067 (11)*	
H12	0.339 (5)	0.615 (3)	0.635 (2)	0.063 (10)*	
H23	0.321 (5)	0.660 (2)	0.384 (2)	0.057 (10)*	
H13	0.514 (5)	0.681 (3)	0.376 (2)	0.067 (9)*	
H2N	0.696 (5)	1.0045 (19)	0.358 (3)	0.043 (9)*	
H1N	0.703 (4)	0.904 (2)	0.4009 (19)	0.042 (7)*	
H4N	0.810 (4)	1.151 (2)	0.548 (2)	0.037 (7)*	
H3N	0.728 (4)	1.156 (2)	0.441 (2)	0.039 (7)*	

### Atomic displacement parameters (Å^2^)

**Table:**

	*U*^11^	*U*^22^	*U*^33^	*U*^12^	*U*^13^	*U*^23^
Sn1	0.03122 (11)	0.01843 (8)	0.02355 (10)	−0.00060 (6)	0.00527 (7)	0.00027 (6)
S1	0.0327 (2)	0.01763 (17)	0.0236 (2)	0.00150 (16)	0.00469 (16)	0.00042 (16)
O3	0.0357 (9)	0.0342 (8)	0.0545 (10)	0.0034 (7)	0.0090 (8)	0.0185 (7)
O2	0.0610 (11)	0.0258 (7)	0.0428 (9)	−0.0059 (7)	0.0273 (8)	−0.0057 (7)
N3	0.0541 (12)	0.0269 (8)	0.0303 (9)	−0.0011 (8)	0.0028 (9)	−0.0010 (7)
N2	0.0540 (12)	0.0272 (8)	0.0251 (9)	−0.0026 (8)	0.0049 (8)	0.0004 (8)
N1	0.0457 (11)	0.0298 (8)	0.0261 (8)	−0.0015 (8)	0.0027 (7)	0.0032 (7)
C6	0.0292 (10)	0.0285 (9)	0.0261 (9)	−0.0004 (7)	0.0038 (7)	0.0007 (7)
C5	0.0507 (14)	0.0320 (10)	0.0444 (13)	0.0013 (9)	0.0053 (10)	0.0127 (10)
C4	0.0657 (18)	0.0578 (16)	0.0242 (11)	−0.0079 (12)	0.0019 (11)	−0.0009 (10)
C1	0.0403 (12)	0.0354 (11)	0.0360 (11)	−0.0087 (9)	0.0002 (9)	0.0015 (9)
O12	0.0907 (14)	0.0267 (7)	0.0301 (8)	0.0010 (8)	0.0200 (8)	−0.0041 (6)
O11	0.0430 (10)	0.0410 (9)	0.0818 (13)	−0.0040 (7)	−0.0171 (9)	0.0149 (9)
O13	0.0646 (10)	0.0178 (6)	0.0325 (7)	0.0041 (6)	0.0137 (7)	0.0008 (5)
O10	0.0517 (10)	0.0287 (7)	0.0543 (10)	0.0027 (7)	0.0277 (8)	0.0093 (7)

### Geometric parameters (Å, °)

**Table:**

Sn1—C1^i^	2.094 (2)	N3—H3N	0.87 (3)
Sn1—C1	2.094 (2)	N2—C6	1.322 (3)
Sn1—O3	2.2140 (16)	N2—H2N	0.79 (3)
Sn1—O3^i^	2.2140 (16)	N2—H1N	0.82 (3)
Sn1—O2	2.2240 (17)	N1—C6	1.331 (3)
Sn1—O2^i^	2.2240 (17)	N1—C5	1.453 (3)
S1—O11	1.4586 (18)	N1—C4	1.457 (3)
S1—O12	1.4654 (16)	C5—H5A	0.9600
S1—O10	1.4710 (16)	C5—H5B	0.9600
S1—O13	1.4725 (14)	C5—H5C	0.9600
O3—H23	0.73 (3)	C4—H4A	0.9600
O3—H13	0.91 (3)	C4—H4B	0.9600
O2—H22	0.79 (4)	C4—H4C	0.9600
O2—H12	0.72 (4)	C1—H1A	0.9600
N3—C6	1.338 (3)	C1—H1B	0.9600
N3—H4N	0.82 (3)	C1—H1C	0.9600
			
C1^i^—Sn1—C1	180.0	H4N—N3—H3N	121 (3)
C1^i^—Sn1—O3	90.52 (8)	C6—N2—H2N	118 (2)
C1—Sn1—O3	89.48 (8)	C6—N2—H1N	122.5 (18)
C1^i^—Sn1—O3^i^	89.48 (8)	H2N—N2—H1N	120 (3)
C1—Sn1—O3^i^	90.52 (8)	C6—N1—C5	122.24 (19)
O3—Sn1—O3^i^	180.000 (1)	C6—N1—C4	121.50 (19)
C1^i^—Sn1—O2	86.95 (9)	C5—N1—C4	116.14 (19)
C1—Sn1—O2	93.05 (9)	N2—C6—N1	121.39 (19)
O3—Sn1—O2	90.16 (7)	N2—C6—N3	118.3 (2)
O3^i^—Sn1—O2	89.84 (7)	N1—C6—N3	120.3 (2)
C1^i^—Sn1—O2^i^	93.05 (9)	N1—C5—H5A	109.5
C1—Sn1—O2^i^	86.95 (9)	N1—C5—H5B	109.5
O3—Sn1—O2^i^	89.84 (7)	H5A—C5—H5B	109.5
O3^i^—Sn1—O2^i^	90.16 (7)	N1—C5—H5C	109.5
O2—Sn1—O2^i^	180.0	H5A—C5—H5C	109.5
O11—S1—O12	109.87 (12)	H5B—C5—H5C	109.5
O11—S1—O10	108.71 (11)	N1—C4—H4A	109.5
O12—S1—O10	109.54 (11)	N1—C4—H4B	109.5
O11—S1—O13	110.27 (10)	H4A—C4—H4B	109.5
O12—S1—O13	108.05 (9)	N1—C4—H4C	109.5
O10—S1—O13	110.40 (9)	H4A—C4—H4C	109.5
Sn1—O3—H23	125 (3)	H4B—C4—H4C	109.5
Sn1—O3—H13	125 (2)	Sn1—C1—H1A	109.5
H23—O3—H13	108 (3)	Sn1—C1—H1B	109.5
Sn1—O2—H22	121 (3)	H1A—C1—H1B	109.5
Sn1—O2—H12	122 (3)	Sn1—C1—H1C	109.5
H22—O2—H12	114 (3)	H1A—C1—H1C	109.5
C6—N3—H4N	116.6 (19)	H1B—C1—H1C	109.5
C6—N3—H3N	118.5 (18)		
			
C5—N1—C6—N2	2.1 (3)	C5—N1—C6—N3	−179.2 (2)
C4—N1—C6—N2	−173.8 (2)	C4—N1—C6—N3	4.9 (3)

Symmetry codes: (i) −*x*+1, −*y*+1, −*z*+1.

### Hydrogen-bond geometry (Å, °)

**Table:**

*D*—H···*A*	*D*—H	H···*A*	*D*···*A*	*D*—H···*A*
O2—H22···O13^ii^	0.79 (4)	1.90 (4)	2.680 (2)	169 (4)
O2—H12···O12^iii^	0.72 (4)	1.91 (4)	2.627 (2)	175 (4)
O3—H23···O11^iv^	0.73 (3)	1.91 (3)	2.629 (3)	168 (3)
O3—H13···O10^v^	0.91 (3)	1.71 (3)	2.615 (2)	172 (3)
N2—H2N···O13^vi^	0.79 (3)	2.10 (3)	2.887 (2)	174 (3)
N3—H3N···O12^vi^	0.87 (3)	2.04 (3)	2.899 (3)	172 (2)
N2—H1N···O10^v^	0.82 (3)	2.14 (3)	2.918 (3)	160 (2)
N3—H4N···O11^vii^	0.82 (3)	2.17 (3)	2.956 (3)	160 (3)

Symmetry codes: (ii) −*x*+3/2, *y*−1/2, −*z*+3/2; (iii) *x*−1, *y*, *z*; (iv) *x*−3/2, −*y*+3/2, *z*−1/2; (v) *x*−1/2, −*y*+3/2, *z*−1/2; (vi) −*x*+2, −*y*+2, −*z*+1; (vii) −*x*+5/2, *y*+1/2, −*z*+3/2.

## References

[bb1] Boye, M. S. & Diasse-Sarr, A. (2007). *C. R. Chim.* **10**, 466–468.

[bb2] Dutrecq, A., Willem, R., Biesemans, M., Boualam, M., Meriem, A. & Gielen, M. (1992). *Main Group Met. Chem.* **15**, 285–291.

[bb3] Farrugia, L. J. (1997). *J. Appl. Cryst.* **30**, 565.

[bb4] Gielen, M. (2005). *Appl. Organomet. Chem.* **19**, 440–450.

[bb5] Molloy, K. C. & Purcell, T. G. (1986). *J. Organomet. Chem.* **312**, 167–176.

[bb6] Molloy, K. C., Quill, K., Cunningham, D., McArdle, P. & Higgins, T. (1989). *J. Chem. Soc. Dalton Trans.* pp 267–273.

[bb7] Nonius (2003). *COLLECT* Nonius BV, Delft, The Netherlands.

[bb8] Otwinowski, Z. & Minor, W. (1997). *Methods in Enzymology*, Vol. 276, *Macromolecular Crystallography*, Part A, edited by C. W. Carter Jr & R. M. Sweet, pp. 307–326. New York: Academic Press.

[bb9] Sheldrick, G. M. (2008). *Acta Cryst.* A**64**, 112–122. 10.1107/S0108767307043930 18156677

[bb10] Westrip, S. P. (2010). *J. Appl. Cryst.* **43**, 920–925.

